# Male and Female Sexual Dysfunction (SD) after Radical Pelvic Urological Surgery

**DOI:** 10.1100/tsw.2006.359

**Published:** 2006-01-19

**Authors:** Paolo Gontero, Francesco Fontana, Ervin Kocjancic, Bruno Frea, Alessandro Tizzani

**Affiliations:** ^1^ Clinica Urologica, Università del Piemonte Orientale, Novara, Italy; ^2^ Clinica Urologica, Università degli Studi di Torino, Torino, Italy

**Keywords:** sexual dysfunction, female, male, radical cystectomy, radical prostatectomy, erectile dysfunction, treatment

## Abstract

Major pelvic urological surgery comprises radical prostatectomy and radical cystectomy in the male patient and radical cystectomy in the female. Postsurgical sexual dysfunction (SD) has been reported with a high prevalence in both sexes and is becoming increasingly important in the patient's view as a result of improved cancer prognosis, refinements in surgical technique, and increased awareness of quality of life aspects that involve sexual satisfaction. The pathophysiology of the problem is essentially related to the disruption of the nerves during the procedure, although a vascular impairment may also be implicated. Nerve-sparing surgery enables the recovery and/or maintenance of sexual functioning in a significant proportion of patients and it is now also adopted for women. Validated questionnaires to assess preoperative baseline sexual function and postoperative outcomes have become available and their use in clinical practice should be promoted. A number of erectile aids are available to treat postsurgical male erectile dysfunction successfully. As far as female SD is concerned, a number of potential treatment options is currently under investigation.

### INTRODUCTION

Major pelvic urological surgery comprises radical prostatectomy and radical cystectomy in the male patient and radical cystectomy in the female. Usually the surgery is carried out for oncological reasons, essentially prostate and bladder cancer, although, particularly in the female, a number of benign conditions (such as interstitial cystitis) may sometimes require bladder removal.

Among the complications of radical urological pelvic surgery, sexual dysfunction (SD) is becoming increasingly important in the patient's view as a result of improved cancer prognosis, refinements in surgical technique, and increased awareness of quality of life aspects that involve sexual satisfaction.

Complete erectile failure has to be expected in nearly all men undergoing radical cystectomy and/or prostatectomy when the procedures are carried out without attempting to spare specific nerve branches that deliver the signal for the onset of erection to the cavernosal bodies. Since the first report in 1982 of a successful surgical technique making it possible to spare the cavernous nerve bundles during radical prostatectomy, the nerve-sparing radical prostatectomy (NSRP) has gained widespread acceptance in the urological community and is nowadays routinely performed in most centers when clinically indicated. The results in terms of potency preservation are not yet satisfactory and vary widely for a number of reasons. Progress in the search for new surgical techniques (i.e., robotic and laparoscopic radical prostatectomy) and new treatment options are being made continuously, as documented by the abundance of literature in this field.

In recent years, a nerve-sparing technique has become available also for male and female radical cystectomy. This approach is still controversial and not routinely adopted in the majority of institutions. Early results in terms of sexual functioning are presently confined to a few reports that have recently been reported in the literature. A new technique of nerve- and seminal-sparing radical cystectomy has also been described for selected patients with excellent outcomes in the maintenance of sexual function. Unlike postradical prostatectomy patients, only scattered data are available on the efficacy of erectile aids in male patients who have undergone radical cystectomy. In addition to the surgical nerve damage, the more aggressive nature of the disease, as well as problems related to the urinary diversion, means that the majority of patients are unlikely to seek help to regain sexual function. The efficacy of medical treatment for female SD has not been demonstrated up to now; hence, the assessment of female sexuality following radical cystectomy is still confined to an investigational level.

The present overview will explore the current knowledge in terms of epidemiology, anatomical and pathophysiological aspects, and treatment options of SD related to females and males who have undergone radical urological surgery.

### BASIC ANATOMY OF PELVIC STRUCTURES RELATED TO SEXUAL FUNCTION THAT CAN BE AFFECTED BY SURGERY

#### In the Male

Essentially, radical urological surgery in the male damages the nerve bundles that govern erection. A more precise knowledge of the neurovascular anatomy of the bladder and the prostate has resulted from cadaveric studies by Walsh and co-workers[1,2]. The neurovascular bundles (NVB) that run posterolateral to the prostate consist of a complex structure related to the vascularization of the outer prostatic portion and to the innervation of the prostate, urethra, and corpora cavernosa. The caudal portion of the pelvic plexus, located at the tip of the seminal vesicles, emanates autonomic fibers which form a dense network[3,4]. Some of them perforate the prostatic capsula to enter the substance of the prostatic gland. These branches are inevitably sacrificed during a nerve-sparing approach, but it is believed that they do not contribute significantly to erectile function. The majority of nerve fibers, known as the cavernous nerves, travel in a direct route from the pelvic plexus toward the posterolateral base of the prostate, gradually coalescing from a group of fibers approximately 12 mm wide to a more organized bundle approximately 6 mm wide at the level of the prostate. At this point, they lie just underneath the lateral pelvic fascia, between the levator fascia and the prostatic fascia (a fibrous layer in direct continuity with the true prostatic capsule). At the level of the membranous urethra, they are located at the 3 o'clock and 9 o'clock positions, just beneath the striated sphincter that at this point surrounds both the urethra and the prostatic apex.

#### In the Female

Female sexual function relies on the integrity of the female external genitalia (which comprises the labia, the clitoris, and the vestibular bulbs) and the internal genitalia (vagina, fallopian tubes, uterus, and ovaries). The clitoris is an erectile organ formed by two erectile bodies fused in the midline, similar to the penile cavernosal bodies. During sexual stimulation, increased blood flow causes clitoris engorgement and subsequent lubrication. The same occurs for the vagina, which becomes engorged during sexual stimulation and forms a plasma trasudate that is critical during the sexual arousal phase[5,6]. The autonomic innervation of the proximal two-thirds of the vagina and clitoris (the equivalent of the male neurovascular bundles) leaves the pelvic plexus and travels within the uterosacral and cardinal ligaments, along with the vessels[7].

### CRITICAL SURGICAL ASPECTS TO PRESERVE SEXUAL FUNCTION

#### Related to Radical Prostatectomy

The first key factor in preserving the patient's ability to regain postoperative spontaneous erections is to ensure the intraoperative preservation of the NVB. Two main surgical approaches to a NSRRP (RRP, radical retropubic prostatectomy) have been described[8]. In the so-called "anatomical technique" first reported by Walsh[2,9,10]**,** the dissection of the nerves is initiated at the apical level with primary isolation of the urethra. Ruckle and Zinke[11] have proposed an alternative technique where the NVB are primarily dissected off the lateral prostate and only subsequently is the urethra transected. Several variants of this lateral approach to the NVB have been described[12,13,14]. Because the cavernosal nerves cannot always be visualized on the dorsolateral prostate surface, Walsh[10] suggests identifying them at the prostatourethral junction after dissecting the apex of the prostate. By contrast, the supporters of the lateral approach to the bundles contend that identifying the nerves at the apex is more difficult[11]. As yet, no study has compared the degree of potency recovery among the different reported nerve-sparing techniques. Outstanding postoperative potency rates varying from 62 to 86% have been reported employing the Walsh nerve-sparing technique, but these seem to be confined to centers of excellence[15,16]. The success rate of the procedure drops to 44% in a large retrospective survey of nonspecialists[17] and falls to 21% in a single institutional prospective series assessing preoperative and postoperative outcomes with validated questionnaires[18]. Discrepancies in the results of NSRRP may be due to a number of factors including age and preoperative sexual function, but there is some evidence that poor outcomes may simply be the result of a suboptimal performance of surgical technique. Recently, Walsh[19] reviewed the videotapes of 62 of his own NSRRP operations. The patient's reported potency rates were correlated with four specific steps of the procedure deemed to be crucial for a correct nerve preservation, namely: V-shaped oversewing of the back bleeders from the proximal dorsal vein on the anterior surface of the prostate, maintenance of a collar of striated sphincter lateral to the urethra while placing the sutures, division of the posterior striated sphincter at its midpoint between the apex and the urethral stump, and achievement of excellent hemostasis at the end of the operation. Notably, in all eight cases where potency had not been regained after 18 months, the technique was judged blindly as less than optimal for all the four steps described above.

Another potential intraoperative key factor affecting the successful recovery of erectile function is the vascular compromise to the cavernosal bodies that follows inadvertent damage of the accessory pudendal arteries. The prevalence of an accessory pudendal branch arising from the inferior vesical or obturator arteries is high and damage of these vessels during the procedure is often unavoidable[20]. These arteries, when present, provide additional blood supply to the cavernosal bodies as evidenced by the finding that as many as 40% of patients have a significant reduction in both the diameter and velocity of blood flow within cavernosal arteries after RRP[21]. Recently, a surgical preservation of assessory pudendal arteries was associated with increased potency rates as well as early recovery of spontaneous erections[22].

#### Related to Male Radical Cystectomy

For accurate nerve preservation during radical cystectomy, Kessler[23] advises NVB preservation dorsolateral to the prostate, as well as more cranially in the angle between the prostate, bladder, and seminal vesicle by ligation and division of the dorsomedial pedicle close to the seminal vesicle.

With the aim of preserving erectile and ejaculatory function, a modified cystectomy that preserves seminal vesicles and the prostatic capsule after transurethral resection[24] or leaving the prostate intact[25] has been successfully reported. Despite the concerns for the oncological control of the disease, excellent results in terms of potency preservation and successful intercourse have been reported with the nerve- and seminal-sparing cystectomy (NSSC)[26,27,28].

#### Related to Female Radical Cystectomy

Surgical factors that can affect female sexuality in regard to cystectomy are (1) the preservation of the nerves (located on the lateral walls of the vagina), (2) the preservation of a stump of distal urethra (in order not to devascularize the clitoris), and (3) the technique of vaginal reconstruction.

Horenblas et al.[25] conducted a prospective trial of modified cystectomy for normal sexual function and anatomic urinary tract reconstruction without oncological concessions. They managed to preserve clitoridal vasculature with the transection of the urethra at the level of the bladder neck and retrograde dissection of the bladder from the anterior vaginal wall. The dissection close to the vaginal wall allows the preservation of both the clitoris and the neurovascular bundle.

Reconstruction of the vagina is achieved by retubularization in a vertical fashion, creating a narrower cylinder or by dissecting the posterior vaginal wall off the rectum to create a flap that is turned downward to form an adequate vaginal caliber, but of decreased depth. A case series reported by Schover and von Eschenbach[29] found a higher rate of sensation of vaginal tightness following radical cystectomy for vertical than for longitudinal retubularization. Of course, the extension of disease influences the amount of upper vaginal wall that has to be removed with possible significant limitations in size after reconstruction with both techniques.

The type of urinary diversion is also a known factor affecting sexuality. An ileal conduit is more likely to disrupt body image than orthotopic ileal neobladder.

### OUTCOME MEASURES OF POSTSURGICAL SEXUAL DYSFUNCTION

#### Questionnaires for Males

The most commonly used questionnaire to assess postsurgical male sexual function is the validated International Index of Erectile Function (IIEF) questionnaire[30]. The following domains are assessed using 15 questions: erectile function (EF), orgasm, sexual desire, intercourse satisfaction, and overall well being. It has now become common practice to administer at least the EF domain before and after radical prostatectomy, whereas this practice is far less common for patients undergoing radical cystectomy. The EF domain of the IIEF has 6 questions; answers are scored from 1 (almost never/never) to 5 (almost always/always), with 0 indicating no sexual activity. Question 3 (Q3) assesses the ability to achieve an erection sufficient for sexual intercourse while question 4 (Q4) assesses the ability to maintain an erection after penetration. The EF domain allows the classification of erectile dysfunction into mild, moderate, or severe if the scores are between 17 and 24, 11 and 16, and <10, respectively. The abridged 5-item version of the IIEF, named the IIEF-5, has been developed to provide a more practical and easy tool to assess EF[31] and its use has gained widespread acceptance in clinical practice.

When a given treatment for postsurgical erectile dysfunction is being assessed in controlled studies, outcome measures, other than the changes in the EF domain scores over the baseline, are currently represented by the 1-item global efficacy question (GEQ) and the Sexual Encounter Profile (SEP) diary. The GEQ asks “Has the treatment you have been taking over the past two or four weeks improved your erections?”. SEP questions include SEP2 (“Were you able to insert your penis into your partner’s vagina?”) and SEP3 (“Did your erection last long enough for you to have successful intercourse?”). An SEP diary is completed by the patient after every attempt at sexual intercourse at baseline and after treatment.

#### Questionnaires for Females

The few studies addressing female sexual function before and after major pelvic surgery have used the Female Sexual Function Index (FSFI), which is one of the few validated tools currently available to screen for female SD[32]. This questionnaire consists of 19 questions concerning 6 domains: desire, arousal, lubrication, orgasm, satisfaction, and pain following vaginal penetration. Individual domain scores are obtained by adding the scores of the individual items in the domain and multiplying the sum by a domain factor.

### PREVALENCE OF SEXUAL DYSFUNCTION ACCORDING TO THE TYPE OF SURGERY (NERVE SPARING VS. NON-NERVE SPARING)

#### Postradical Prostatectomy Sexual Dysfunction

Patients with a bilateral transection of the NVB, as occurs in non-nerve–sparing radical prostatectomy (NNSRP), are not expected to regain a spontaneous erection[33]. By contrast, the proportion of individuals who experience complete recovery of EF following bilateral anatomical preservation of the NVB during NSRP remains a matter of debate, but probably does not occur in more than about 50% of patients overall. A further 25% reduction is expected if only one NVB can be spared at the time of surgery. As already mentioned, age and preoperative sexual functioning are also independent predictors of postoperative erectile dysfunction. The chances of potency recovery decrease with an increase in age at the time of the operation. The excellent results in the series reported by Walsh refer mainly to patients younger than 65[34]. Noh et al.[35] reported that all patients younger than 50 in his series regained postoperative sexual function compared with only 38% older than 70. Similarly, patients with some degree of erectile dysfunction prior to the procedure are more likely to develop severe erectile dysfunction postoperatively[36]. Another key factor is the timing for potency recovery. At 3 months postoperatively, only 38% of patients in the Walsh series were able to perform unassisted sexual intercourse, whereas potency had been regained by 86% at 18 months[19]. Differences in surgical technique, outcome measurements, and follow-up duration have been suggested as explanations for these discrepancies, but none of the reported studies mentions whether the patients had undergone specific “erectile rehabilitation treatment” (i.e., the use of erectile aids) during the follow-up period. It is of note, however, that the only series where patients were counseled to start a pharmacological erectile treatment as early as the second postoperative month gave the best results of potency recovery at 24 months[34].

The most recent series has concentrated on the results of nerve preservation following laparoscopic radical prostatectomy showing success rates comparable to open surgery[37] In the study by Katz et al.[38], patients were deliberately asked not to undertake any postoperative erectile rehabilitation and, despite that, a high-potency preservation rate was observed.

### Postradical Cystectomy Sexual Dysfunction in Men

In 1984, Walsh and Mostwin[39] were the first to show that their nerve-sparing technique resulted in an increased postoperative potency in individuals who had undergone radical cystoprostatectomy, with an 82% potency preservation rate at 1 year in 11 patients. In 1990, Brendler et al.[40] reported that 27 out of 52 patients (52%) who had undergone nerve-sparing cystoprostatectomy had recovered erections 1 year after the procedure. Since these reports, other studies have reported similar rates of potency after nerve-sparing radical cystectomy, ranging from 42–71%[41,42]. In a recent series[43], the IIEF-5 was administered to 49 preoperatively potent subjects who had undergone cystoprostatectomy at a median follow-up of 47 months. The mean postoperative scores were significantly reduced compared to the preoperative values, with 86% of patients unable to achieve vaginal penetration after surgery. Of note, only 6 out of 16 (37%) patients who had undergone a nerve-sparing procedure had erections sufficient for vaginal penetration.

Sexual function was assessed at 2.5 years follow-up in a large series of 331 patients who had undergone radical cystectomy and neobladder, which involved monolateral or bilateral nerve sparing in 77% of the cases[23]. Although validated questionnaires were not employed and preoperative sexual function was not reported, 73.4% reported no erections in the postoperative period. The overall 2-year recovery rate of erectile function (with or without the use of erectile aids) was only 31%, although the majority of them had reduced erections (that is, erections not always firm enough for sexual intercourse).

If we take into account that these data come from centers of excellence, then we would expect even worse figures in the average success rate of a nerve-sparing radical cystectomy throughout worldwide institutions.

By contrast, the NSSC has become highly acceptable to patients in view of the early recovery of postoperative sexual function that occurs within 1 or 2 months and the high rate of potency sparing. In the series reported by Colombo et al., 21 out of 22 patients regained sexual function after the procedure and showed postoperative IIEF scores not dissimilar to the preoperative ones[28]. However, the oncological safety of this surgical technique is still debatable.

### Postradical Cystectomy Sexual Dysfunction in Women

Recently, two studies have addressed sexual function after female radical cystectomy. Zippe et al.[43], using a modified 10-item version of the FSFI[44], reported a significant decrease in the mean preoperative scores compared with the baseline values. The most common symptoms reported by the patients included diminished ability to achieve orgasm in 45% of cases, decreased lubrication in 41%, decreased sexual desire in 37%, and dyspareunia in 22%. Only 13 (48%) of the 27 preoperatively sexually active patients were able to have successful vaginal intercourse at a mean interval of 24.2 months after the procedure. No statistically significant difference in the FSFI scores was found between the different types of urinary diversion (orthotopic neobladder vs. cutaneous continent pouch vs. ileal conduit diversion).

A similar decrease in postoperative FSFI scores was reported in 29 patients who had undergone radical cystectomy and orthotopic ileal neobladder, with only 40% being sexually active at a time interval of 1 to 17 years after surgery[45].

## PATHOPHYSIOLOGY OF SEXUAL DYSFUNCTION FOLLOWING PELVIC UROLOGICAL SURGERY

### In Males

In male patients undergoing radical pelvic urological surgery without sparing of the nerves, a complete irreversible cessation of any form of spontaneous erectile activity is expected to occur. Spontaneous EF is also absent for the majority of patients in the early postoperative period of a nerve-sparing radical prostatectomy or cystectomy while a progressive return is observed over a 2-year period in a variable proportion of cases. This is likely to be due to the so-called “neuropraxia” phenomenon, i.e., a temporary deficit of the spared cavernous nerves due to surgical manipulation that would abolish any form of erection for a variable period of time.

The partial pressure of oxygen in penile blood is only 25–40 mmHg during flaccidity, whereas it reaches 90–100 mmHg during erection[46]. The low oxygen tensions in a constantly flaccid penis may switch on (i.e., initiate) severe fibrotic changes in the cavernosal tissue. In a recent experimental model, penile tissue from rats that had undergone bilateral incision of cavernosal nerves 3 months earlier showed a significant overexpression of hypoxia-related substances like TGF-beta and collagen I and III compared with the same tissue from a control group[47]. These results have been confirmed by Hu et al.[48] employing the same experimental model: at 15 weeks from surgery, rats that had undergone bilateral transection of both cavernous nerves showed nearly twice the TGF-beta expression both at the mRNA and protein level compared with the sham-operated control group. Consequently, the ratio of cavernosal smooth muscle/collagen fibers was significantly lower in the neurectomy than in the control group. This is in keeping with previous findings of an increased collagen synthesis in human corpus cavernosum smooth muscle cells in culture from hypoxia induced TGF-beta[49]. Additionally, User et al.[50] documented a significant apoptosis in the subalbugineal smooth muscle cells following bilateral neurotomy of the rat penis. In contrast to healthy men, where nocturnal penile erections can maintain a high level of penile blood partial oxygen pressure that may inhibit the synthesis of collagen induced by TGF-beta, in postradical prostatectomy and cystectomy patients, fibrotic penile changes occur even when the cavernous nerves have been preserved during surgery. Histomorphometric studies have shown that when a high proportion of trabecular smooth muscle is replaced by collagen, the caverno-occlusive mechanism is lost with subsequent venogenic erectile dysfunction.

How can fibrosis be prevented? Early use of proerectile aids (PDE5 inhibitors such as sildenafil or intracavernous PGE1 injections) may guarantee sufficient cavernosal oxygen tension, thereby reducing the risk of cavernous occlusive dysfunction. A penile hemodynamic study on NSRP patients who had no pharmacological support in the initial 12 months after surgery revealed a progressive incidence of venous leakage varying from 14% at 4 months to 50% at more than 12 months[51]. Similarly, in a study by Montorsi et al.[52], 53% of patients who did not self-inject with PGE1 in the first 4 months after surgery had a color doppler diagnosis of venous leakage vs. only 17% of the treatment group. Schwartz et al.[53] evaluated the cavernosal smooth muscle content in 40 patients before undergoing NSRP and after 6 months of postoperative treatment with sildenafil. A statistically significant increase in mean smooth muscle content was observed in the group of patients receiving 100 mg sildenafil compared with those treated with 50 mg sildenafil. These findings corroborate the hypothesis that erectile rehabilitation prevents the occurrence of vasculogenic erectile dysfunction during the process of nerve healing after NSRP, probably by reducing the postoperative hypoxia induced cavernosal fibrosis.

As regards patients who had radical prostatectomy with “non-nerve–sparing intent”, a 60% failure to achieve an erection sufficient for sexual intercourse occurs if intracavernous PGE is started 4 months after the operation, whereas the likelihood of regaining a functional pharmacological erection reaches 70% when the injection is administered within 3 months of the operation[54]. Color doppler ultrasound studies suggest that treatment failures are mainly due to cavernous occlusive dysfunction, although an arteriogenic factor is also implicated. As already discussed above, a significant proportion of patients show a reduction in both the diameter and velocity of blood flow within cavernosal arteries after RRP[21], probably due to intraoperative damage of the accessory pudendal artery that provides additional blood supply to the cavernosal bodies[20].

### In Females

Sympathetic and parasympathetic fibers innervating the clitoris and the vagina are usually transected during a non-nerve–sparing cystectomy resulting in a lack of blood engorgement of these organs with a subsequent severe loss of lubrication that causes a sexual arousal disorder. Fibrotic changes of the clitoridal erectile tissue following denervation are likely to occur via the same pathophysiological mechanism described for the male, leading to interference with normal clitoridal relaxation and dilation responses to sexual stimulation. Loss of clitoridal corporal smooth muscle with replacement by fibrous connective tissue has so far been demonstrated by histomorphometric studies on clitoridal erectile tissue of females with decreased vaginal and clitoridal engorgement from arterial insufficiency[55]. Vascular insufficiency to the vagina and the clitoris may follow hysterectomy (a normal procedure during radical cystectomy) through the ligation of the uterine pedicle that provides some arterial supply to those organs. Other factors, such as estrogen deprivation (when ovaries are removed) and resection of a significant portion of the vagina, may further contribute to the complexity of symptoms that describe postcystectomy female sexual dysfunction.

### PATIENT'S CONCERN ABOUT SEXUAL FUNCTION FOLLOWING PELVIC UROLOGICAL SURGERY

Studies addressing quality of life issues following oncological urological pelvic surgery have highlighted the negative impact of postsurgical SD.

#### Quality of Life Impact of Sexual Function after Radical Prostatectomy

In one study, patients with erectile dysfunction after radical prostatectomy were found to have better disease-specific quality of life than a matching group of erectile dysfunction patients with no history of prostate cancer[56]. The former are probably more likely to accept with stoicism SD acquired after having received a potentially curative treatment for cancer. In another study, patients who were more concerned about loss of sexual function after radical prostatectomy would not trade much of their current life span to regain sexual function[57]. However, the fact that patients can live with their SD and would elect to have the same treatment for prostate cancer does not mean that their quality of life is totally unaffected. By comparing sexual function after non-nerve–sparing and nerve-sparing surgery, Gralnek et al.[58] reported that the latter had significantly better postoperative quality of life scores in regard to both sexual and physical function. More recent studies have explored the meaning of "sexual dysfunction" after radical prostatectomy and noted that several aspects of health-related quality of life were affected including sexual intimacy, everyday interaction with women, and men's perception of their masculinity[59]. The negative impact of SD on quality of life may become even more marked a longer time after the operation. In the study by Penson et al.[60], SD was an independent determinant of worse general health-related quality of life 2 years after the primary treatment for prostate cancer.

#### Quality of Life Impact of Sexual Function after Radical Cystectomy

The few studies addressing quality of life after radical cystectomy have highlighted the significant negative impact of SD on overall well being. Interestingly enough, in the study by Schover et al.[61], it was reported that although 90% of men experienced some degree of erectile dysfunction following radical cystectomy, 50% remained sexually active, at least using noncoital stimulation. For 95 French patients alive 5 years after surgery who completed a functional assessment of cancer therapy (FACT) questionnaire, cystectomy clearly led to more negative scores in sexuality-related questions, impotency and loss of sexual interest being the main complaints[62]. Based on a self-completed questionnaire, lack of sexual activity significantly reduced the quality of life of cystectomy patients as compared to patients who had received more conservative treatment (radiotherapy) for bladder cancer[63]. In another study of 224 patients completing quality of life questionnaires, SD was identified as the most common complaint together with problems related to urine diversion. Of interest, penile prosthesis placement was associated with better satisfaction. The authors concluded that physicians should discuss SD as a long-term correlate of radical cystectomy and offer the option of erectile aids[64].

### TREATMENT OPTIONS AND THEIR EFFICACY FOR SEXUAL DYSFUNCTION AFTER PELVIC UROLOGICAL SURGERY

#### General Concepts

Current treatment options for male erectile dysfunction are (1) the oral PDE5 inhibitors molecules sildenafil, vardenafil, and tadalafil; (2) intracavernous injections (ICI) of vasoactive agents (such as PGE1); (3) the vacuum pump; and (4) different models of penile prosthesis that are surgically implanted[65]. All these erectile aids are usually adopted after radical pelvic urological surgery with variable degrees of success depending on the patient's motivation to resume sexual activity, treatment compliance, and the type of surgery (nerve sparing vs. non-nerve sparing). As an example, the PDE5 inhibitors, usually considered as the first-line treatment option, are thought to be highly ineffective after a non-nerve–sparing procedure since no amplification of the nitric oxide (NO) cascade occurs after the administration of a PDE5 inhibitor in the absence of nitric oxide (NO) release from intact cavernosal nerve fibers. By contrast, the same drugs can produce a significant response rate when the nerves have been carefully preserved.

Previous questionnaire-based surveys have pointed out that the majority of patients with postoperative erectile dysfunction do not use any erectile aids, although the reason for this could not be identified[58,66]. Additionally, patient's compliance in using erectile aids seems to be low, as documented by the relatively high rates of postprostatectomy patients who discontinue treatment for sexual dysfunction[67]. We will briefly summarize the rationale and efficacy of erectile aids when used either as a temporary prophylaxis (while awaiting the return of spontaneous erection after a nerve-sparing procedure) or as a permanent treatment when surgical denervation has occurred.

As far as females are concerned, pharmacological treatment is investigational at present with phase III trials currently in progress. Recent studies have demonstrated the efficacy of sildenafil in treating female SD associated with aging and menopause[68]. The use of erectile aids as a treatment option for postsurgical female SD remains a totally unexplored field.

#### Pharmacological Prophylaxis after Nerve-Sparing Radical Cystoprostatectomy

Nocturnal penile tumescence is still severely impaired 8 months after a NSRP as a consequence of neuropraxia of the cavernosal nerves[69]. Early intake of a PDE5 type inhibitor at bedtime has been recommended with the aim of making nocturnal erections more potent. In the preliminary study by Padma-Nathan et al.[70], patients who were potent preoperatively were randomized after NSRP to sildenafil or a placebo for 36 weeks. Those who regained sexual function after 9 months of treatment (27%) also had better nocturnal erections recorded a year after the operation. It is possible that sildenafil, as well as the other currently available PDE5 type inhibitors such as vardenafil and tadalafil, may not be so effective in the early phase of nerve healing as documented by the clinical inefficacy of sildenafil in the first 9 months after a NSRP[71].

Three monthly intracavernous injections with PGE1, starting the first postoperative month, significantly enhanced subsequent response to sildenafil as compared with sildenafil alone started at the fourth postoperative month. At the 6 month follow-up, 82% of patients in the combination arm responded to subsequent sildenafil vs. only 52% in the sildenafil-only arm[72]. Intracavernosal therapy has been found to produce a good erectile response in non-nerve–sparing patients and, therefore, it may be the treatment of choice in the early postoperative period following a nerve-sparing procedure. Similarly, the use of a vacuum constrictor device may facilitate early sexual intercourse and the potentially early return of natural erections, although no controlled study has been carried out to test this hypothesis. From the point of view of patient's tolerability, a trend favoring ICI treatment rather than a vacuum device was reported in a randomized trial on men with erectile dysfunction due to other causes[73].

Based on the little data available, either intracavernosal injections or a vacuum device should be offered as a first-line option for the first few months after the procedure as their mechanism of action does not require intact neural tissue for erection. Thereafter sildenafil, or an equivalent PDE inhibitor therapy, may be a reasonable choice for those patients who can achieve at least a partial erection.

#### Curative Treatment after Non-Nerve–Sparing Radical Cystoprostatectomy

Studies of the efficacy of oral treatment after non-nerve–sparing procedures are based on small series on postprostatectomy patients reporting a response rate to sildenafil ranging from 0%[71] to 15%[74]. Intracavernous pharmacotherapy still remains the mainstay treatment option for patients who fail to recover spontaneous erections after radical prostatectomy and high response rates have been reported employing mixtures of vasoactive agents[75,76]. In a recent study, we recorded a 60% response rate for PGE (which is the only drug licensed for intracavernous injections), although injections were judged more painful than the vacuum device. Surprisingly, the percentage of patients choosing injections for domestic use was as low as 16% and comparable to the vacuum device[77]. In general, the overall response rate to the available nonsurgical treatment options may be somewhat influenced by individual motivation, but it should not be expected to go beyond 50 to 60%. Half of the patients are likely to discontinue the treatment within a year. Treatment of non-nerve–sparing erectile dysfunction therefore remains a difficult and frustrating task for the urologist. European Guidelines state that for patients who fail pharmacological therapy or who prefer a permanent solution to their problem, surgical implantation of a prosthesis may be considered[65].

In the absence of studies specifically addressing the use of erectile aids after radical cystectomy, the same or even worse figures than those reported for radical prostatectomy have to be expected.

### CONCLUSIONS

Sexual dysfunction following radical pelvic urological surgery adversely affects the overall quality of life of both men and women. The prevalence of erectile dysfunction in males approaches 100% when a non-nerve–sparing procedure is performed and, given the disappointing results of the erectile aids currently available, indications for nerve-preserving surgery should be extended whenever possible. Rehabilitative treatment should be offered to all patients undergoing a nerve-sparing procedure in order to maximize the spontaneous recovery of EF and to prevent carvernosal fibrosis. Little is known about the extent of female SD after radical pelvic surgery, but initial studies seem to suggest that it is considerable. Future research should concentrate on refinements of nerve-sparing radical cystectomy techniques, sexual counseling, and the development of new treatment options.

## References

1. Schlegel, P.N. and Walsh, P.C. (1987) Neuroanatomical approach to radical cystoprostatectomy with preservation of sexual function. *J. Urol*. **138(6),** 1402–1406.
2. Walsh, P.C. and Donker, P.J. (1982) Impotence following radical prostatectomy: insight into etiology and prevention. *J. Urol*. **128(3),** 492–497.
3. Lue, T.F., Zeineh, S.J., Schmidt, R.A., and Tanagho, E.A. (1984) Neuroanatomy of penile erection: its relevance to iatrogenic impotence. *J. Urol*. **131(2),** 273–280.
4. Lepor, H. et al. (1985) Precise localization of the autonomic nerves from the pelvic plexus to the corpora cavernosa: a detailed anatomical study of the adult male pelvis. *J. Urol*. **133(2),** 207–212.
5. Berman, J.R., Berman, L.A., and Kanaly, K.A. (2003) Female sexual dysfunction: new perspectives on anatomy, physiology, evaluation and treatment. *Eau Update Series* **1(3),** 166–177.
6. Weber, A.M., Walters, M.D., Schover, L.R., and Mitchinson, A. (1995) Sexual function in women with uterovaginal prolapse and urinary incontinence. *Obstet. Gynecol*. **85(4),** 483–487.

7. Sjoberg, I. (1992) The vagina: morphological, functional and etiological aspects. *Acta Obstet. Gynecol. Scand.* **71**, 84.
8. Gillitzer, R. and Thuroff, J.W. (2003) Technical advances in radical retropubic prostatectomy techniques for avoiding complications. II. Vesico-urethral anastomosis and nerve-sparing prostatectomy. *BJU Int*. **92(2),** 178–184.
9. Walsh, P.C. (1998) Anatomic radical prostatectomy: evolution of the surgical technique. *J. Urol*. **160(6 Pt 2),** 2418–2424.
10. Walsh, P.C. (2002) Anatomic radical retropubic prostatectomy. In *Campbell's Urology*. 8th ed. Vol. 4. Walsh, P.C., Retik, A.B., Vaughan, E.D., Jr., and Wein, A.J., Eds. WB Saunders, Philadelphia. pp. 3107–3129.
11. Ruckle, H.C. and Zincke, H. (1995) Potency-sparing radical retropubic prostatectomy: a simplified anatomical approach. *J. Urol*. **153(6),** 1875–1877.
12. Klein, E.A., Kupelian, P.A., Tuason, L., and Levin, H.S. (1998) Initial dissection of the lateral fascia reduces the positive margin rate in radical prostatectomy. *Urology* **51(5),** 766–773.
13. Jarow, J.P. and Trock, B.J. (2002) Anatomic radical prostatectomy: does initial dissection of lateral fascia affects outcomes? *J. Urol*. **167(Suppl),** A 1363.
14. Fernandez, S. (2002) Experience with water-jet dissection in nerve sparing radical prostatectomy. *J. Urol*. **167(Suppl),** A 1338.
15. Walsh, P.C. (2000) Radical prostatectomy for localised prostate cancer provides durable cancer control with excellent quality of life: a structured debate. *J. Urol*. **163(6),** 1802–1807.
16. Catalona, W.J. and Basler, J.W. (1993) Return of erections and urinary continence following nerve sparing radical retropubic prostatectomy. *J. Urol*. **150**, 905–909.
17. Standford, J.L. et al. (2000) Urinary and sexual function after radical prostatectomy for clinically localised prostate cancer: the prostate cancer outcomes study. *JAMA* **283(3),** 354–360.
18. Talcott, J.A. et al. (1997) Patient-reported impotence and incontinence after nerve-sparing radical prostatectomy. *J. Natl. Cancer Inst*. **89(15),** 1117–1123.
19. Walsh, P.C., Marschke, P., Ricker, D., and Burnett, A.L. (2000) Use of intraoperative video documentation to improve sexual function after radical retropubic prostatectomy. *Urology* **55**, 62–67.
20. Droupy, S., Hessel, A., Benoit, G., Blanchet, P., Jardin, A., and Giuliano, F. (1999) Assessment of the functional role of accessory pudendal arteries in erection by transrectal color doppler ultrasound. *J. Urol*. **162**, 1987.
21. Aboseif, S., Shinohara, K., Breza, J., Benard, F., and Narayan, P. (1994) Role of penile vascular injury in erectile dysfunction after radical prostatectomy. *BJU Int*. **73**, 75.
22. Rogers, C.G., Trock, B.P., and Walsh, P.C. (2004) Preservation of accessory pudendal arteries during radical retropubic prostatectomy: surgical techniques and results. *Urology* **64(1),** 148–151.
23. Kessler, T.M., Burkhard, F.C., Perimenis, P., Danuser, H., Thalmann, G.N., Hochreiter, W.W., and Studer, U.E. (2004) Attempted nerve sparing surgery and age have a significant effect on urinary continence and erectile function after radical cystoprostatectomy and ileal orthotopic bladder substitution. *J. Urol*. **172(4 Pt 1),** 1323–1327.
24. Colombo, R., Bertini, R., Salonia, A., Da Pozzo, L.F., Montorsi, F., Brausi, M., Rossigno, M., and Rigatti, P. (2001) Nerve and seminal sparing radical cystectomy with orthotopic urinary diversion for select patients with superficial bladder cancer: an innovative surgical approach. *J. Urol*. **165(1),** 51–55.
25. Horenblas, S., Meinhardt, W., Ijzerman, W., and Moonen, L.F. (2001) Sexuality preserving cystectomy and neobladder: initial results. *J. Urol*. **166(3),** 837–840.
26. Vallancien, G., Abou El Fettouh, H., Cathelineau, X., Baumert, H., Fromont, G., and Guillonneau, B. (2002) Cystectomy with prostate sparing for bladder cancer in 100 patients: 10-year experience. *J. Urol*. **168(6),** 2413–2417.
27. Meinhardt, W. and Horenblas, S. (2003) Sexuality preserving cystectomy and neobladder (SPCN): functional results of a neobladder anastomosed to the prostate. *Eur. Urol*. **43(6),** 646–650.
28. Colombo, R., Bertini, R., Salonia, A., Naspro, R., Ghezzi, M., Mazzoccoli, B., Deho', F., Montorsi, F., and Rigatti, P. (2004) Overall clinical outcomes after nerve and seminal sparing radical cystectomy for the treatment of organ confined bladder cancer. *J. Urol*. **171(5),** 1819–1822.
29. Schover, L.R. and von Eschenbach, A.C. (1985) Sexual function and female radical cystectomy: a case series. *J. Urol*. **134**, 465.
30. Rosen, R.C., Riley, A., and Wagner, G. (1997) The International Index of Erectile Function (IIEF): a multidimensional scale for assessment of erectile dysfunction. *Urology* **49**, 822–825.
31. Rosen, R.C., Cappelleri, J.C., Smith, M.D., Lipsky, J., and Pena, B.M. (1999) Development and evaluation of an abridged, 5-item version of the International Index of Erectile Function (IIEF-5) as a diagnostic tool for erectile dysfunction. *Int. J. Impot. Res.* **11(6),** 319–326.
32. Rosen, R., Brown, C., Heiman, J., Leiblum, S., Meston, C., Shabsigh, R., Ferguson, D., and D'Agostino, R., Jr. (2000) The Female Sexual Function Index (FSFI): a multidimensional self-report instrument for the assessment of female sexual function. *J. Sex Marital Ther*. **26(2),** 191–208.
33. Sachs, B.D. and Liu, Y.C. (1991) Maintenance of erection of penile glans, but not penile body, after transection of rat cavernous nerves. *J. Urol*. **146(3),** 900–905.
34. Walsh, P.C. et al. (2000) Patient-reported urinary continence and sexual function after anatomic radical prostatectomy. *Urology* **55**, 58–61.
35. Noh, C., Kshirsagar, A., and Mohler, J.L. (2003) Outcomes after radical retropubic prostatectomy. *Urology* **61(2),** 412–416.

36. McCullough, A.R. (2001) Prevention and management of erectile dysfunction following radical prostatectomy. *Urol. Clin. North Am.* **28,** 613–627.
37. Hara, I., Kawabata, G., Miyake, H., et al. (2003) Comparison of quality of life following laparoscopic and open prostatectomy for prostate cancer. *J. Urol*. **169(6),** 2045–2048.
38. Katz, R., Salomon, L., Hoznek, A., et al. (2002) Patient reported sexual function following laparoscopic radical prostatectomy. *J. Urol*. **168(5),** 2078–2082.
39. Walsh, P.C. and Mostwin, J.L. (1984) Radical prostatectomy and cystoprostatectomy with preservation of potency. Results using a new nerve-sparing technique. *Br. J. Urol*. **56(6),** 694–697.
40. Brendler, C.B., Steinberg, G.D., Marshall, F.F., Mostwin, J.L., and Walsh, P.C. (1990) Local recurrence and survival following nerve-sparing radical cystoprostatectomy. *J. Urol*. **144(5),** 1137–1140.
41. Venn, S.N., Popert, R.M., and Mundy, A.R. (1998) 'Nerve-sparing' cystectomy and substitution cystoplasty in patients of either sex: limitations and techniques. *Br. J. Urol*. **82(3),** 361–365.
42. Koraitim, M. and Khalil, R. (1992) Preservation of urosexual functions after radical cystectomy. *Urology* **39(2),** 117–121.
43. Zippe, C.D., Raina, R., Shah, A.D., Massanyi, E.Z., Agarwal, A., Ulchaker, J., Jones, S., and Klein, E. (2004) Female sexual dysfunction after radical cystectomy: a new outcome measure. *Urology* **63(6),** 1153–1157.
44. Kaplan, S.A., Reis, R.B., Kohn, I.J., Ikeguchi, E.F., Laor, E., Te, A.E., and Martins, A.C. (1999) Safety and efficacy of sildenafil in postmenopausal women with sexual dysfunction. *Urology* **53(3),** 481–486.
45. Volkmer, B.G., Gschwend, J.E., Herkommer, K., Simon, J., Kufer, R., and Hautmann, R.E. (2004) Cystectomy and orthotopic ileal neobladder: the impact on female sexuality. *J. Urol*. **172(6 Pt 1),** 2353–2357.
46. Kim, N., Vardi, Y., Padma-Nathan, H., Daley, J., Goldstein, I., and Saenz de Tejada, I. (1993) Oxygen tension regulates the nitric oxide pathway. Physiological role in penile erection. *J. Clin. Invest*. **91(2),** 437–442.
47. Leungwattanakij, S., Bivilacqua, T.J., Usta, M.F., et al. (2003) Cavernous neurotomy causes hypoxia and fibrosis in rat corpus cavernosum. *J. Androl*. **24(2),** 239–245.
48. Hu, W., Hu, L., Li, S., Zheng, X., and Tian, B. (2004) Expression of transforming growth factor-β1 in penile tissue from rats with bilateral cavernosal nerve ablation. *BJU Int*. **94**, 424–428.
49. Moreland, R.B., Traish, A., McMillin, M.A., Smith, B., Goldstein, I., and Saenz de Tejada, I. (1995) PGE1 suppresses the induction of collagen synthesis by transforming growth factor-beta 1 in human corpus cavernosum smooth muscle. *J. Urol*. **153(3 Pt 1),** 826–834.
50. User, H.M., Hairston, J.H., Zelner, D.J., McKenna, K.E., and McVary, K.T. (2003) Penile weight and cell subtype specific changes in a post-radical prostatectomy model of erectile dysfunction. *J. Urol*. **169(3),** 1175–1179.
51. Mulhall, J.P., Slovick, R., Hotaling, J., et al. (2002) Erectile dysfunction after radical prostatectomy: hemodynamic profiles and their correlation with the recovery of erectile function. *J. Urol*. **167(3),** 1371–1375.
52. Montorsi, F., Guazzoni, G., Ferini Strambi, L., et al. (1997) Recovery of spontaneous erectile function after nerve-sparing radical retropubic prostatectomy with and without early intracavernous injections of alprostadil: results of a prospective, randomized trial. *J. Urol*. **158**, 1408–1410.
53. Schwarts, E.J., Wong, P., and Graydon, R.J. (2004) Sildenafil preserves intracorporeal smooth muscle after radical retropubic prostatectomy. *J. Urol*. **171(2),** 771–774.
54. Gontero, P., Fontana, F., Bagnasacco, A., et al. (2003) Is there an optimal timing for intracavernous PGE1 rehabilitation following nonnerve sparing radical prostatectomy? Results from an hemodynamic perspective study. *J. Urol*. **169(6),** 2166–2169.
55. Park, K., Goldstein, I., Andry, C., Siroky, M.B., Krane, R.J., Azadzoi, K.M. (1997) Vasculogenic female sexual dysfunction: the hemodynamic basis for vaginal engorgement insufficiency and clitoral erectile insufficiency. *Int. J. Impot. Res.* **9(1),** 27–37.
56. Penson, D.F., Latini, D.M., Lubeck, D.P., Fallace, K., Henning, J.M., and Lue, T. (2003) Is quality of life different for men with erectile dysfunction and prostate cancer compared to men with erectile dysfunction due to other causes? Results from the exceed data base. *J. Urol*. **169**, 1458–1461.
57. Smith, D.S., Krygiel, J., Nease, R.F., Sumner, W., Jr., and Catalona, W.J. (2002) Patient preferences for outcomes associated with surgical management of prostate cancer. *J. Urol*. **167**, 2117–2122.
58. Gralnek, D., Wessells, H., Cui, H., and Dalkin, B.L. (2000) Differences in sexual function and quality of life after nerve sparino and nonnerve sparino radical retropubic prostatectomy. *J. Urol*. **163**, 1166–1170.
59. Bokhour, B.G., Clark, J.A., and Inui, T.S. (2001) Sexuality after treatment for early prostate cancer: exploring the meanings of "erectile dysfunction". *J. Gen. Intern. Med*. **16**, 649–655.
60. Penson, D.F., Feng, Z., Kuniyuri, A., et al. (2003) General quality of life 2 years following treatment for prostate cancer: what influences outcomes? Results from the prostate cancer outcome study. *J. Clin. Oncol*. **21(6),** 1147–1154.
61. Schover, L.R., Evans, R., and von Eschenbach, A.C. (1986) Sexual rehabilitation and male radical cystectomy. *J. Urol*. **136(5),** 1015–1017.
62. Matsuda, T., Aptel, I., Exbrayat, C., and Grosclaude, P. (2003) Determinants of quality of life of bladder cancer survivors five years after treatment in France. *Int. J. Urol*. **10(8),** 423–429.
63. Caffo, O., Fellin, G., Graffer, U., and Luciani, L. (1996) Assessment of quality of life after cystectomy or conservative therapy for patients with infiltrating bladder carcinoma. A survey by a self-administered questionnaire. *Cancer* **78(5),** 1089–1097.

64. Hart, S., Skinner, E.C., Meyerowitz, B.E., Boyd, S., Lieskovsky, G., and Skinner, D.G. (1999) Quality of life after radical cystectomy for bladder cancer in patients with an ileal conduit, cutaneous or urethral kock pouch. *J. Urol*. **162(1),** 77–81.
65. Wespes, E., Amar, E., Hatzichristou, D., Montorsi, F., Pryor, J., and Vardi, Y. (2002) European Association of Urology. Guidelines on erectile dysfunction. *Eur. Urol*. **41(1),** 1–5.
66. Perez, M.A., Meyerowitz, B.E., Lieskovsky, G., Skinner, D.G., Reynolds, B., and Skinner, E.C. (1997) Quality of life and sexuality following radical prostatectomy in patients with prostate cancer who use or do not use erectile aids. *Urology* **50(5),** 740–746.
67. Raina, R., Lakin, M.M., Thukral, M., et al. (2003) Long-term efficacy and compliance of intracorporeal (IC) injection for erectile dysfunction following radical prostatectomy: SHIM (IIEF-5) analysis. *Int. J. Impot. Res*. **15(5),** 318–322.
68. Berman, J.R., Berman, L.A., Lin, H., Flaherty, E., Lahey, N., Goldstein, I., and Cantey-Kiser, J. (2001) Effect of sildenafil on subjective and physiologic parameters of the female sexual response in women with sexual arousal disorder. *J. Sex Marital Ther*. **27(5),** 411–420.
69. Fraiman, M.C., Lepor, H., and McCullough, A.R. (1999) Nocturnal penile tumescence activity in 81 patients presenting with erectile dysfunction (ED) after nerve sparing radical prostatectomy. *J. Urol*. **161(4S),** Suppl. 179.
70. Padma-Nathan, H., Mc Cullough, A.R., Giuliano, F., Toler, S., Wohlhuter, C., and Shpilsky, A. (2003) Postoperative nightly administration of sildenafil citrate significantly improves the return of normal spontaneous erectile function after bilateral nerve-sparing radical prostatectomy. *J. Urol*. **169(Suppl),** 1402.
71. Zagaja, G.P., Mhoon, D.A., Aikens, J.E., and Brendler, C.B. (2000) Sildenafil in the treatment of erectile dysfunction after radical prostatectomy. *Urology* **56(4),** 631–634.
72. Montorsi, F., Salonia, A., Barbieri, L., et al. (2002) The subsequent use of I.C. alprostadil and oral sildenafil is more efficacious than sildenafil alone in nerve sparing radical prostatectomy. *J. Urol*. **167(4),** Suppl. 279.
73. Soderdahl, D.W., Thrasher, J.B., and Hansberry, K.L. (1997) Intracavernosal drug-induced erection therapy versus external vacuum devices in the treatment of erectile dysfunction. *BJU Int*. **79(6),** 952–957.
74. Zippe, C.D., Jhaveri, F.M., Klein, E.A., Pasqualotto, F.F., Kedia, A., Agarwal, A., Montague, D.K., and Lakin, M.M. (2000) Role of Viagra after radical prostatectomy. *Urology* **55(2),** 241–245.
75. Baniel, J., Israilov, S., Segenreich, E., and Livne, P.M. (2001) Comparative evaluation of treatments for erectile dysfunction in patients with prostate cancer after radical retropubic prostatectomy. *BJU Int*. **88**, 58–61.
76. Dennis, R.L. and McDougal, W.S. (1988) Pharmacological treatment of erectile dysfunction after radical prostatectomy. *J. Urol*. **139**, 775–779.
77. Gontero, P., Fontana, F., Zitella, A., Montorsi, F., and Frea, B. (2005) A prospective evaluation of efficacy and compliance with a multistep treatment approach for erectile dysfunction in patients after non-nerve sparing radical prostatectomy. *BJU Int*. **95(3),** 359–365.

### BIOSKETCH

The scientific activity of **Dr. Paolo Gontero** in the last few years has been largely devoted to the field of urology oncology and oncology andrology. As regards the latter, a few studies appearing in major urological reviews have assessed the use of different erectile aids in non-nerve–sparing radical prostatectomy men and overviewed the techniques of nerve-sparing radical prostatectomy.
